# Immunoprotective effect and mechanism of rEg.P29 against CD4 ^+^ T cell-deficient mice with *Echinococcus multilocularis* infection: Protection and mechanism of rEg.P29 against *E*. *multilocularis*

**DOI:** 10.3724/abbs.2023282

**Published:** 2023-12-28

**Authors:** Ming Li, Yazhou Zhu, Zihua Li, Jiahui Song, Wei Zhao

**Affiliations:** 1 Department of Hepatobiliary Surgery Ningxia Medical University General Hospital Yinchuan 750004 China; 2 Department of Medical Immunology and Pathogen Biology Ningxia Medical University Yinchuan 750004 China; 3 Ningxia Key Laboratory of Prevention and Control of Common Infectious Disease Ningxia Medical University Yinchuan 750004 China

**Keywords:** CD4 ^+^ T cell-deficient mice, rEg.P29, *Echinococcus multilocularis*

## Abstract

Alveolar echinococcosis (AE) is a zoonotic parasitic disease caused by infection with the larval stage of *Echinococcus multilocularis* and a major challenge to human public health. Vaccines are the most effective way to prevent and control infectious diseases. We previously revealed that the *Echinocuccus granulosus* recombinant protein P29 is a good vaccine candidate against *E*. *granulosus*. However, the protective and immunological mechanism of rEg.P29 against *E*. *multilocularis* remain unclear. In this study, CD4 ^+^ T cell-deficient mice are transferred with spleen CD4 ^+^ T cells isolated from wild-type mice and subjected to rEg.P29 immunization, and then these immunized mice are infected with *E*. *multilocularis*. The cyst inhibition rate is calculated by weighing the body and cyst weights. The level of antibody is detected by ELISA. Flow cytometry is used to detect the level of IFN-γ production by CD4 ^+^ T and CD8 ^+^ T cells. The cytokines in culture supernatant are detected by ELISA. The expressions of CD44 and CD62L on memory T cells are determined by flow cytometry. The results show the cyst inhibition rate is 41.52% after adoptive transfer of CD4 ^+^ T cells. Furthermore, the levels of IgG, IgM, IgA and IgE in serum are significantly increased compared with those in the PBS group. The IFN-γ-secretion by CD8 ^+^ T cells and the level of IFN-γ in culture supernatant are obviously increased; and the number of CD4 ^+^ T cells is increased, but the number of IFN-γ producing CD4 ^+^ T cells has no significant difference compared with PBS group. In addition, the number of CD44 ^+^CD62L ^‒^CD8 ^+^ memory T cells in the spleen is significantly increased, while the number of CD44 ^‒^CD62L ^+^ CD8 ^+^ memory T cells is not significantly altered. Collectively, rEg.P29 can alleviate *E*. *multilocularis* infection by inducing humoral immune responses and CD8 ^+^ T cell responses.

## Introduction

Alveolar echinococcosis (AE) is a sufferable chronic parasitic disease caused by the intrahepatic tumor-like growth of the metacestode of *Echinococcus multilocularis* [ 1– 3], and is also a zoonotic disease that seriously endangers human health and life [4]. Humans can serve as aberrant intermediate hosts, getting infection by ingestion of eggs by chance [5]. The eggs enter the liver through blood circulation and parasitize the liver tissue. Therefore, AE mainly originates from the liver. Hepatic AE shows infiltrative growth in the host liver, and can cause liver injury, hepatic coma, and portal hypertension in patients. In addition to the liver tissue, AE can also be found in the brain and other organisms, which can seriously threaten a patient’s life [ 6, 7]. At present, surgery with drug therapy is the preferred AE treatment choice. However, surgical resection of this hydatid disease is often incomplete, causing patients to relapse easily [8]. Drug therapy has many side effects, and its efficacy varies significantly among different patients [9]. Furthermore, other preventive measures have no distinct effects on AE. Hence, it is very important to determine a timely and effective approach to prevent *E*. *multilocularis* infection.

Vaccination against *E*. *multilocularis* may be an alternative or feasible treatment method [10]. In recent years, a large number of *E*. *multilocularis* antigens have been found to be able to induce adaptive immune responses and play vital roles both in intermediate and definitive hosts, such as EG95 [11] and 14-3-3 [12]. rEg.P29 is a novel 29 kDa antigen of *Echinococcus granulosus* [13]. Our previous study found the protective efficacy of rEg.P29 in sheep and mouse models with secondary infection of *E*. *granulosus* were 94.5% and 96.6%, respectively [ 14, 15]. In addition, rEg.P29 can induce mice to produce high levels of IgG, IgM, IgE and IgA antibodies, and significantly increase IFN-γ secretion from CD4 ^+^ T and CD8 ^+^ T cells [16]. These data suggested that rEg.P29 can induce high levels of humoral and cellular immune responses. However, how rEg.P29, as a novel antigen of *E*. *granulosus*, prevents *E*. *multilocularis* infection is unclear.

In this study, we constructed a mouse model of rEg.P29 immunization combined with *E*. *multilocularis* infection, clarified the protective effect of rEg.P29 against *E*. *multilocularis* infection in CD4 ^+^ T cell-deficient mice.

## Materials and Methods

### Antigen purification and endotoxin removal

The expression and purification of rEg.P29 were performed following previously published methods [17]. In brief, in order to express rEg.P29, *E*. *coli* was induced in the presence of 50 μg/mL isopropyl β-D-1-thiogalactopyranoside (IPTG; Invitrogen, Carlsbad, USA) for 10 h at 37°C. Then, according to the manufacturer’s instructions for the His Purification kit (Merck, Darmstadt, Germany), the rEg.P29 was purified. The purified rEg.P29 was identified by 10% SDS-PAGE, and the concentration of rEg.P29 was measured using a CBA kit (KeyGEN Biotech, Nanjing, China). Finally, the endotoxin was removed using an endotoxin removal kit (GenScript Biotech Corporation, Piscataway, USA), and the endotoxin concentration was detected using an endotoxin detection kit (GenScript Biotech Corporation).

### Isolation and viability identification of protocephalic larvae

Protoscoleces (PSCs) were isolated from *E*. *multilocularis*-infected gerbils. Briefly, cysts were isolated from gerbils and stripped tissue components of mice. Sterile PBS buffer was used to wash cyst samples. Then, the cyst tissue was shredded and placed on a 300-mesh iron filter for grinding and rinsed with PBS while grinding. The filtrate was filtered through a 200-mesh sieve and naturally settled for 5 min. The supernatant was discarded, and the sample was washed 2 times with PBS. PSCs were stained with 1% eosin (Beyotime, Shanghai, China) to identify their viability. PSCs inocula with a viability over 95% confirmed by 1% eosin exclusion were used for portal injection in mice.

### Identification of CD4 ^+^ T cell-deficient mice

DNA extraction was performed using the DNA extraction kit (TIANGEN, Beijing, China) according to the manufacturer’s instructions. In brief, 1‒2 mm of mouse tail was cut off, and DNA was extracted. Then, polymerase chain reaction (PCR) was performed in a reaction mixture (25 μL) containing 12.5 μL of PCR master mix (Vazyme, Nanjing, China), 1.5 μL of template DNA, 1 μL of each primer, and 9 μL of nuclease-free water (TIANGEN). The sequences of the forward primers were 5′-CTAGCATGAATGAGGAGGATGGG-3′ and 5′-CACTCAGCCTTGTTCTCTGACC-3′, the sequences of the reverse primers were 5′-CTGGCAGGTCTTCTTCTCACTG-3′, and PCR amplification was undertaken using the following protocol: 94°C for 3 min; 94°C for 30 s (denaturation), 60°C for 35 s (annealing), 72°C for 35 s (extending), for 35 thermal cycles; 72°C for 5 min, hold at 4°C. The amplified products were analyzed and confirmed by agarose gel electrophoresis.

### CD4 ^+^ T cell isolation from wild-type mice and adoptive transfer

Spleen cells of wild-type mice were isolated immediately by pushing the spleen through a 70-μm strainer in Hank’s balanced salt solution, followed by Ficoll-Hypaque (Tianjin HaoYang Biological Manufacture, Tianjin, China) density gradient centrifugation at 450 *g* for 20 min. Mononuclear cells were collected, washed twice with buffer, and divided it into three groups in 300 μL/tube. CD4 ^+^ T cells were sorted using CD4 ^+^ T cell magnetic bead isolation kit (Miltenyi Biotec, Bergisch Gladbach, Germany) according to manufacturer’s instructions. Then, staining for APC-labelled anti-CD4 (BD Biosciences, San Jose, USA) markers was performed, including incubation at 4°C in the dark for 30 min. After washing twice, the cells were suspended in 2 mL of PBS with 10% FBS and sorted by flow cytometry with a BD FACSAria™ III flow cytometer (BD, Franklin Lakes, USA). The purity of CD4 ^+^ T cells was > 99%. Finally, CD4 ^+^ T cells were collected for use in subsequent experiments.

### Animal immunization and infection

**Figure 1:**
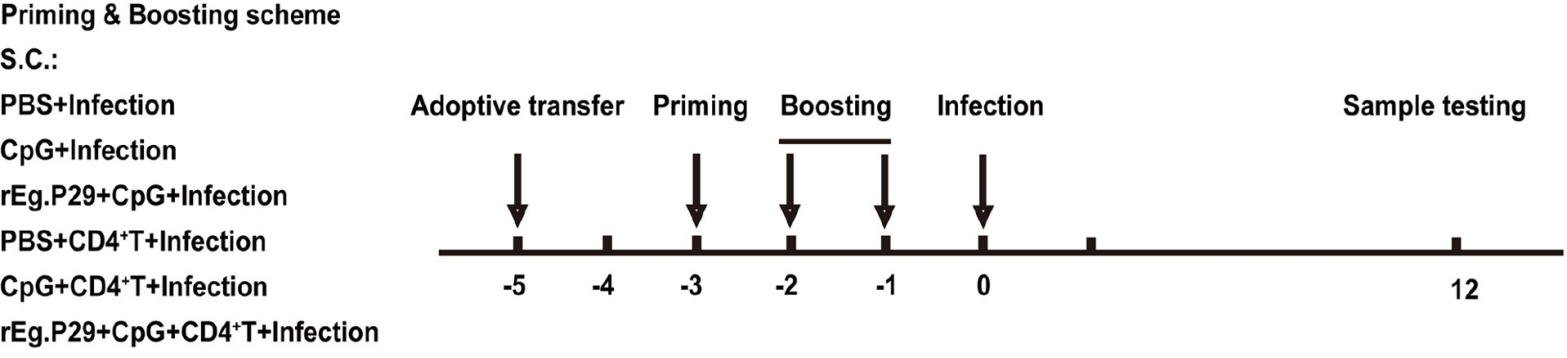
Figure 1 Scheme of immunization and sampling protocol Splenic CD4+ T cells from wild-type mice were transferred via the tail vein to CD4+ T cell-deficient mice (‒5) and were immunized following the prime-boost scheme. Two boosts (‒2 and ‒1) were performed in the process. After one week, 2000 PSCs/200 μL were intraperitoneally injected into each mouse (0). Spleen samples and serum samples were collected and tested at the 12th week (12).

Wild-type C57BL/6 mice were obtained from the Experimental Animal Center of Ningxia Medical University. C57BL/6N-Cd4emlcyagen (strain number: KOCMP-12504-Cd4-B6N-VA) was purchased from Cyagen Biosciences (Cyagen, Suzhou, China). The study was approved by the Experimental Animal Ethics Committee of Ningxia Medical University (No. KYLL-2021-765), and experiments were carried out in strict accordance with national and institutional guidelines. Eighteen identified homozygous mice were randomly divided into 6 groups: PBS (subcutaneously injected with 100 μL PBS buffer)+Infection (intraperitoneally injected with 2000 PSCs/200 μL PBS); CpG (subcutaneously injected with 20 μg CpG ODN 1826)+Infection; rEg.P29 (subcutaneously injected with 20 μg rEg.P29)+CpG+Infection; PBS+CD4 ^+^T (adoptive transfer of wild-type mouse CD4 ^+^ T cells)+Infection; CpG+CD4 ^+^T+Infection; rEg.P29+CpG+CD4 ^+^T+Infection. For subcutaneous immunization, the emulsified mixture was suspended in PBS and injected into the lower quadrant of the abdomen (100 μL/mouse). The immunization and infection scheme is shown in Figure 1.

### Sample collection and cell culture

Three months later, the mice were anaesthetized with isoflurane, and blood samples and spleens were collected from each mouse. Spleen cells were isolated immediately by pushing the spleen through a 70-μm strainer in balanced buffer (PBS with 10% calf serum), followed by Ficoll-Hypaque (Haoyang, Tianjin, China) density gradient centrifugation at 450 g for 20 minutes. Then, monocytes were collected and washed twice with buffer. Finally, monocytes were resuspended at a final concentration of 2×10 ^6^ cells/mL in complete RPMI 1640 medium (HyClone, Logan, USA) including 10% calf serum (Gemini Bio, West Sacramento, USA), 100 U/mL penicillin/streptomycin (Pricella, Wuhan, China), 2 mM L-glutamine (Nuowei Biotec, Beijing, China), and 50 μM 2-mercaptoethanol (Gibco, Grand Island, USA).

### ELISA

A total of 200 μL of cell suspension was plated into each well of a round-bottom 96-well plate and stimulated with or without rEg.P29 protein (10 μg/mL) in the presence of anti-CD28 antibody (1 μg/mL) at 37°C with 5% CO _2_ for 3 days. Cytokines (IFN-γ, TNF-α, IL-2, IL-4, and IL-6) in the supernatants were detected using BD OptEIA Mouse ELISA Sets (BD Biosciences, San Jose, USA) according to the manufacturer’s instructions. H _2_SO _4_ (1 M) was used to stop the reaction, and the absorbance of each well was measured at 450 nm with a microplate reader (Thermo Fisher, Waltham, USA). According to standard serial dilutions of cytokines, the concentrations of each sample were calculated.

For antibody detection of each sample, according to the requirements of the reagent instructions, the antibodies in the serum were detected by enzyme-linked immunosorbent assay (ELISA). In brief, 10 μg/mL rEg.P29 was coated on the ELISA plate and incubated at 4°C overnight. The plates were washed five times with PBST (PBS containing 0.05% Tween 20) and blocked with 5% skim milk powder in PBST at 37°C for 1 h. After five times wash with PBST, the plates were incubated with mouse serum (1:500) in 5% skim milk powder in PBST for 2 h and washed five times with PBST for 3 min. Horseradish peroxidase (HRP)-conjugated anti-mouse IgM, IgG, IgA (100 μL each; Abcam, Scottsdale, USA) and IgE (100 μL; Invitrogen) were added to the plates and incubated at 37°C for 1 h. After washing, 100 μL TMB Single-Component Substrate solution (Solar bio, Beijing, China) was added and incubated for 8–10 min, and the reaction was stopped by addition of 2 M H _2_SO _4_. The absorbance was measured at 450 nm with an ELISA plate reader (Thermo Fisher, Waltham, USA).

### Flow cytometry

To measure the level of intracellular cytokines, a 2×10 ^6^ cells/mL cell suspension was diluted to 1×10 ^6^ cells/mL with RPMI 1640 and stimulated with or without rEg.P29 (10 μg/mL) in the presence of anti-CD28 antibody (1 μg/mL) at 37°C with 5% CO _2_ for 20 h. In addition, brefeldin A (Sigma-Aldrich, St Louis, USA) was added to the culture at a concentration of 10 μg/mL. The cells were washed twice with buffer 1 (PBS with 10% FCS). Then, fluorochrome-conjugated monoclonal antibodies (mAb) were used to stain for phenotyping for 30 min at 4°C in the dark. Then, the cells were washed with buffer, fixed with 4% paraformaldehyde and permeabilized with buffer 2 (PBS with 10% FCS and 0.1% saponin) overnight at 4°C. Intracellular cytokines were detected by fluorochrome-conjugated mAbs staining for 30 min at 4°C in the dark. Cells were washed with buffer and measured using a FACS Celesta (BD Biosciences) for data collection. Data were analyzed using Flow Jo 10 (Tree Star, San Carlos, USA).

For memory cell detection, cells were washed with buffer and then stained with CD3, CD8, CD62L and CD44 mAbs (BD Biosciences) for 30 min at 4°C in the dark. The cells were washed twice with buffer and measured with a FACS Celesta flow cytometer (BD Biosciences) for data collection. Data were analyzed using Flow Jo 10 (Tree Star).

### Statistical analysis

All data were analyzed using statistics software SPSS 20.0 (IBM, Armonk, USA) and GraphPad Prism 8.0 (GraghPad Software, La Jolla, USA). Unpaired Student’s *t*-test was used for comparisons between two groups, and one-way or two-way ANOVA was used for comparisons among more than two groups. Data are presented as the mean or the mean±SD. *P*<0.05 was considered statistically significant.

## Results

### CD4 ^+^ T cell preparation and identification

**Figure 2:**
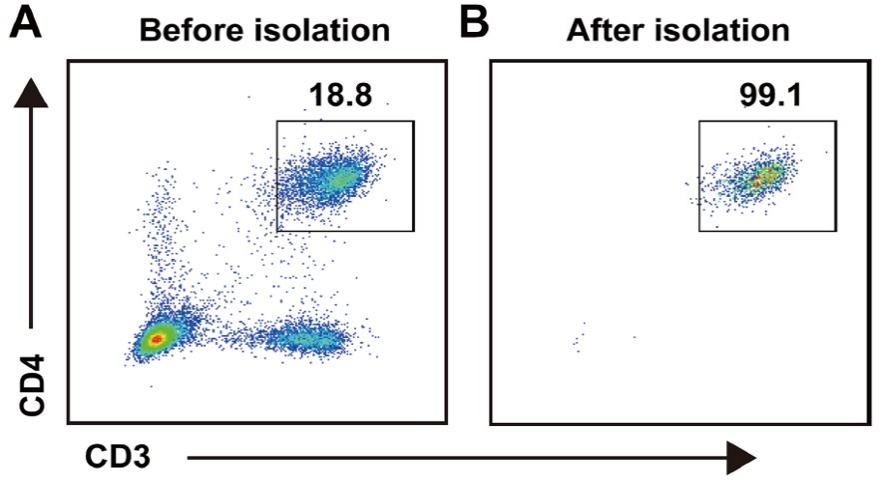
Figure 2 Flow cytometry was used to detect the proportion of CD4 ^+^ T cells before and after sorting The spleen was collected from wild-type C57BL/6 N mice, and lymphocytes were isolated. LCM was used to sort CD4+ T cells. (A) The proportion before isolation. (B) The proportion after isolation.

The CD4 ^+^ T cells of the spleen were sorted by using a magnetic bead isolation kit, and the purity of CD4 ^+^ T cells was detected by flow cytometry. The results showed that the proportion of CD4 ^+^ T cells in the spleen was 18.8% before isolation, and was increased to 99.4% after isolation, as shown in Figure 2A,B. These data confirmed that the sorted CD4 ^+^ T cells can be used for adoptive transfer.

### rEg.P29 alleviated *E*. *multilocularis* infection in CD4 ^+^ T cell-deficient mice

Our previous data showed the immune protection of rEg.P29 against *E* . *granulosus*-infected mice and sheep was 94.3% and 98.6% [ 14, 15], respectively. However, the protection and mechanism of rEg.P29 against *E*. *multilocularis* remain unclear. Here, CD4 ^+^ T cell-deficient mice with or without CD4 ^+^ T cells from wild-type mice were immunized with rEg.P29 and then subjected to *E*. *multilocularis* infection. The cyst inhibition rate analysis showed the immune protection of rEg.P29 against *E*. *multilacularis* infection in CD4 ^+^ T cell-deficient mice was 35.74% compared with the control; after adoptive transfer of CD4 ^+^ T cells from wild-type mice, the protection of rEg.P29 against *E*. *multilocularis* infection in CD4 ^+^T cell-deficient mice was 41.52% compared with the control ( Table 1).

**Table:** **Table 1** The body and cyst weights of each group

Group	Body weight (g)	Cyst weight (g)	Cyst inhibition rate ^a^
PBS+Infection	29.81±4.45	5.99±2.55	
CpG+Infection	36.66±1.86	8.62±0.90	
rEg.P29+CpG+Infection	28.38±2.59	3.85±3.33*	35.74%
PBS+CD4 ^+^T+Infection	41.96±1.61	9.10±5.30	
CpG+CD4 ^+^T+Infection	38.92±4.52	8.37±1.22	
rEg.P29+CpG+CD4 ^+^T+Infection	35.64±2.86	5.34±1.44 ^#^	41.52%

^a^Cyst inhibition rate (%)=[(cyst weight of PBS group‒cyst weight of rEg.P29 immunization group)/cyst weight of PBS group]×100%. * *P*<0.05 vs PBS+Infection group; ^#^ *P*<0.05 vs PBS+CD4 ^+^T+Infection group.

Twelve weeks after infection, the autopsy results showed that the size and weight of cyst in the abdominal cavity of rEg.P29+Infection group were significantly decreased compared with those in the PBS+Infection group and CpG+Infection group ( Supplementary Figure S1A–C). After transfer of adoptive CD4 ^+^ T cells, the size and weight of cyst in the abdominal cavity of CD4 ^+^T+rEg.P29+CpG+Infection group were also reduced compared with those in the CD4 ^+^T+PBS+Infection group and CD4 ^+^ T+CpG+Infection group ( Supplementary Figure S1D–F). These data suggested that rEg.P29 alleviated *E* . *multilocularis* infection in CD4 ^+^ T cell-deficient mice.

### rEg.P29 induced an antibody response in CD4 ^+^ T cell-deficient mice with *E*. *multilocularis* infection

**Figure 3:**
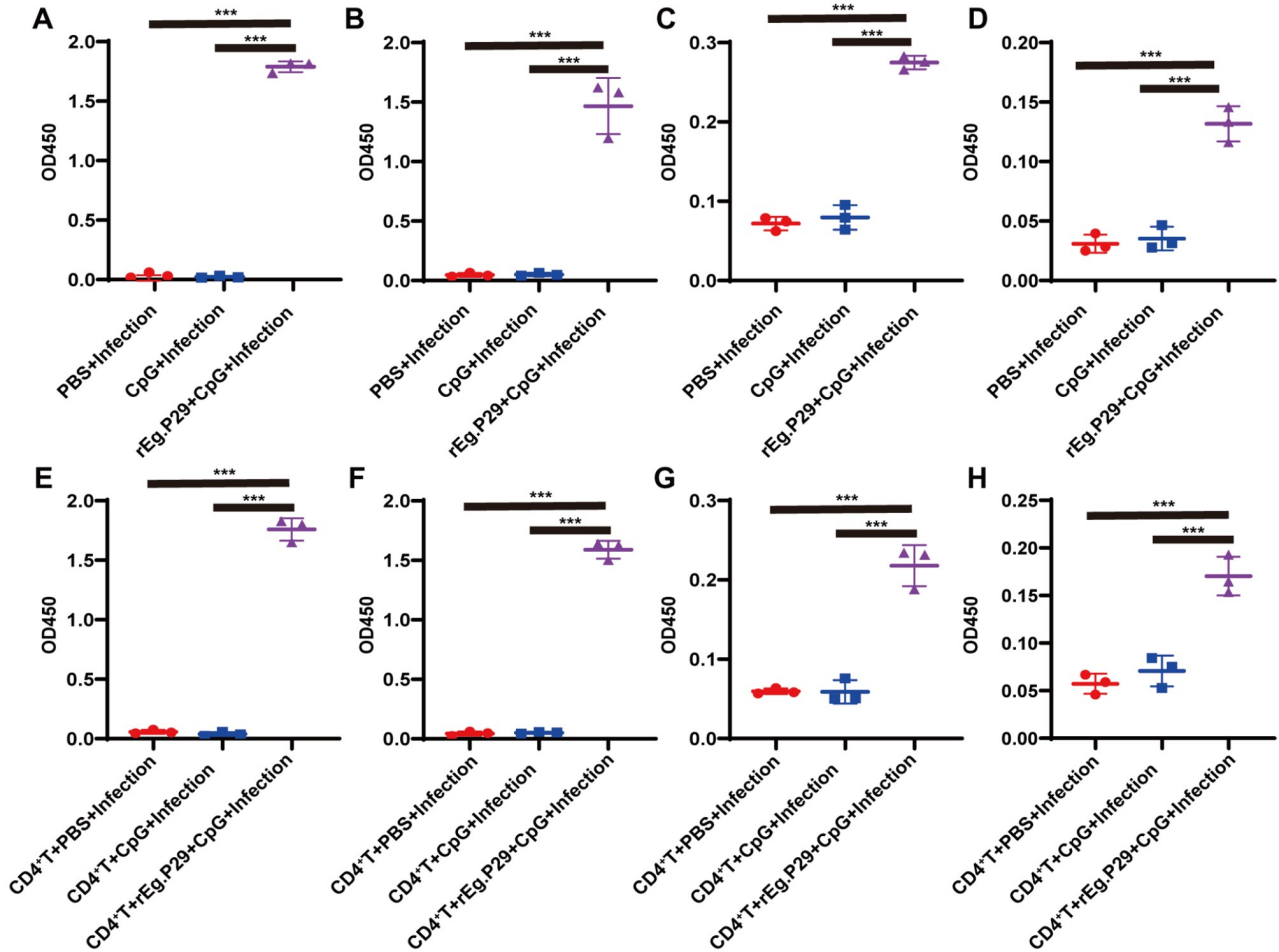
Figure 3 The level of antibody in serum was detected by ELISA Twelve weeks after E. multilocularis infection, serum was collected to determine the antibody level. (A‒D) Before adoptive transfer, the change in antibodies in serum was detected. (E-H) After adoptive transfer, the change in antibodies in serum was detected. (A,E) The level of IgG in serum. (B,F) The level of IgM in serum. (C,G) The level of IgA in serum. (D,H) The level of IgE in serum. ***P<0.001.

To further reveal the mechanism of the protective effect, we analyzed the antibody response against rEg.P29 in CD4 ^+^ T cell-deficient mice. The antibody subtypes IgG, IgM, IgA and IgE in serum samples were tested by ELISA. As shown in Figure 3, the levels of rEg.P29-specific IgG, IgM, IgA and IgE were significantly increased in the rEg.P29+CpG+Infection compared with those in the PBS group and CpG group. The levels of rEg.P29-specific IgG, IgM, IgA and IgE of CpG+Infection group showed no significant difference compared with those of the PBS group ( Figure 3A‒D). After adoptive transfer of CD4 ^+^ T cells, the levels of IgG, IgM, IgA and IgE in serum of rEg.P29+CpG+Infection were significantly increased compared with those of the CD4 ^+^T+PBS+Infection and CD4 ^+^T+CpG+Infection group. The levels of IgG, IgM, IgA and IgE of the CD4 ^+^T+CpG+Infection group were not significantly different from those of the CD4 ^+^ T+PBS group ( Figure 3E,F). These data suggested that rEg.P29-induced specific antibodies could prevent *E*. *multilocularis* infection in CD4 ^+^ T cell-deficient mice.

### rEg.P29 induced CD8 ^+^ T cells response in CD4 ^+^ T cell-deficient mice, but did not induce CD4 ^+^ T cell response during *E*. *multilocularis* infection

**Figure 4:**
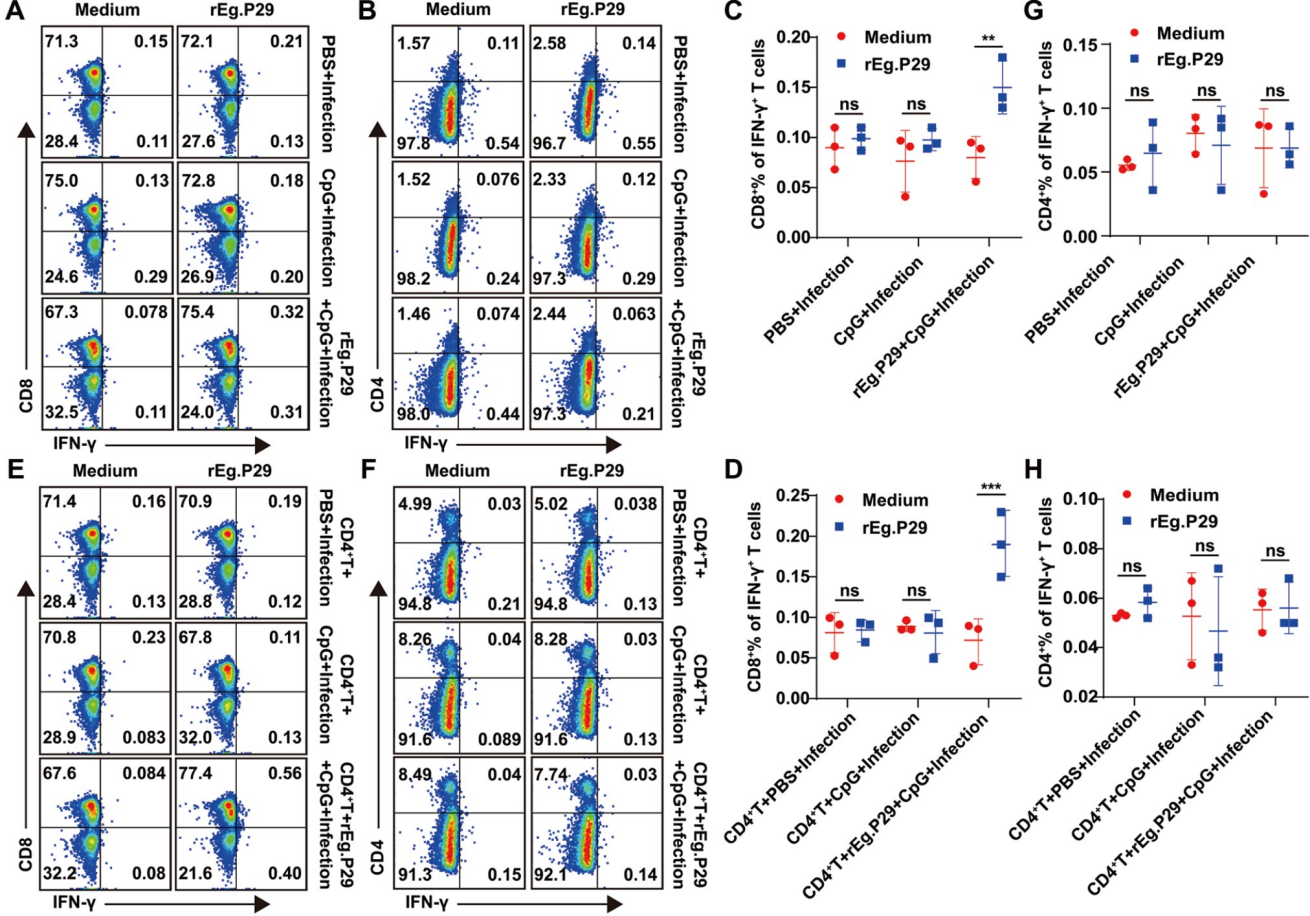
Figure 4 The expression of IFN-γ in CD4 ^+^ T cells and CD4 ^+^ T cells before and after adoptive transfer of CD4 ^+^ T cells from wild-type mice was detected by flow cytometry (A,E) The expression of IFN-γ in CD8+ T cells before or after adoptive transfer was detected by flow cytometry. (B,F) The expression of IFN-γ in CD4+ T cells before or after adoptive transfer was detected by flow cytometry. (C,D) Frequencies of IFN-γ-producing CD8+ T cells in each group are shown. (G,H) Frequencies of IFN-γ-producing CD4+ T cells in each group are shown. **P<0.01. ns, not significant.

To understand the roles of the cellular response in the process of *E* . *multilocularis* infection, specific cytokines produced by lymphocytes in the spleen of CD4 ^+^ T cell-deficient mice in each group before or after adoptive transfer of CD4 ^+^ T cells were detected to identify the level and type of cellular response against *E*. *multilocularis*. The results showed that IFN-γ secretion by CD8 ^+^ T cells was significantly increased in the rEg.P29+CpG+Infection group with rEg.P29 stimulation when compared with that without stimulation. The IFN-γ secretion by CD8 ^+^ T cells of the PBS+Infection group and CpG+Infection group with rEg.P29 stimulation showed no difference compared with that without stimulation. However, the IFN-γ secretion by CD4 ^+^ T cells of the rEg.P29+CpG+Infection group with rEg.P29 stimulation showed no difference compared with that without stimulation, and the number of CD4 ^+^ T cells was not different compared with that of the PBS+Infection group and CpG+Infection group ( Figure 4A‒D). After adoptive transfer of CD4 ^+^ T cells, IFN-γ secretion by CD8 ^+^ T cells of CD4 ^+^T+rEg.P29+CpG+Infection group with rEg.P29 stimulation was significantly increased when compared with that without stimulation. The IFN-γ secretion by CD8 ^+^ T cells of the CD4 ^+^T+PBS+Infection group and CD4 ^+^T+CpG+Infection group with rEg.P29 stimulation showed no difference compared with that without stimulation. In addition, the number of CD4 ^+^ T cells was significantly increased, but the IFN-γ secretion by CD4 ^+^ T cells of CD4 ^+^T+rEg.P29+CpG+Infection group with rEg.P29 stimulation showed no difference compared with that without stimulation ( Figure 4E‒H).

**Figure 5:**
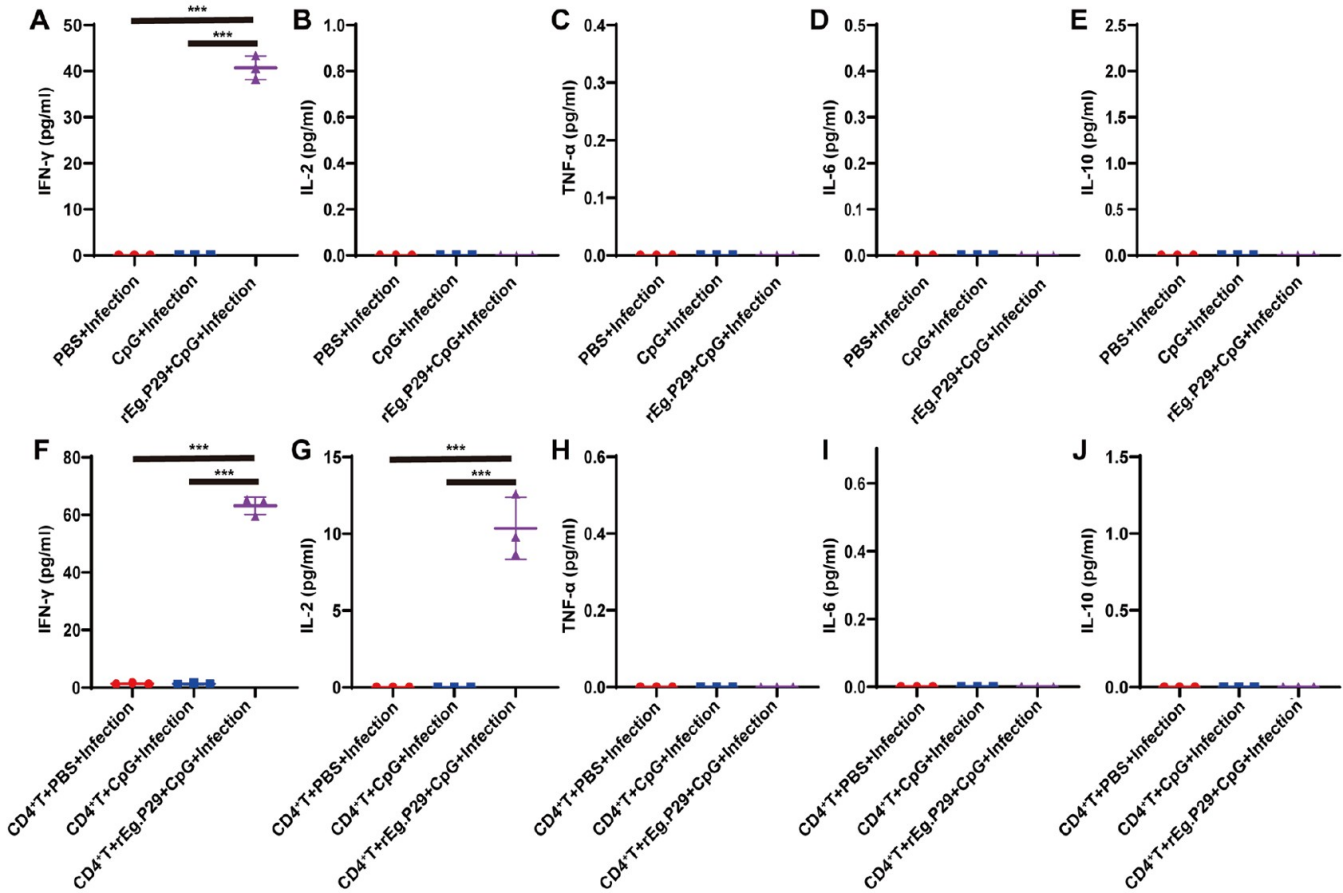
Figure 5 Cytokines were detected in the culture supernatant by ELISA Twelve weeks after E. multiloculair infection, lymphocytes were collected and stimulated with rEg.P29 protein, ELISA analysis of the changes in cytokines of each group before or after adoptive transfer. (A‒E) Cytokines (IFN-γ, IL-2, TNF-α, IL-6 and IL-10) were detected before adoptive transfer. (F‒J) Cytokines were tested after adoptive transfer. ***P<0.001.

Moreover, the cytokines in the culture supernatant were detected by ELISA. The results showed that the level of IFN-γ of rEg.P29+CpG+Infection group was significantly increased, but the levels of IL-2, IL-6, TNF-α, and IL-10 showed no difference compared with those of the PBS+Infection group and CpG+Infection group ( Figure 5A‒E). After adoptive transfer of CD4 ^+^ T cells, the levels of IFN-γ and IL-2 of CD4 ^+^T+rEg.P29+CpG+Infection group were significantly increased, but the levels of TNF-α, IL-6, and IL-10 showed no difference compared with those of the CD4 ^+^ T+PBS+Infection group and CD4 ^+^ T+CpG+Infection ( Figure 5F‒J). These data suggested that rEg.P29 can induce CD8 ^+^ T cell response to alleviate *E*. *multilocularis*.

### rEg.P29 induced CD8 ^+^ memory T cells in the spleen

**Figure 6:**
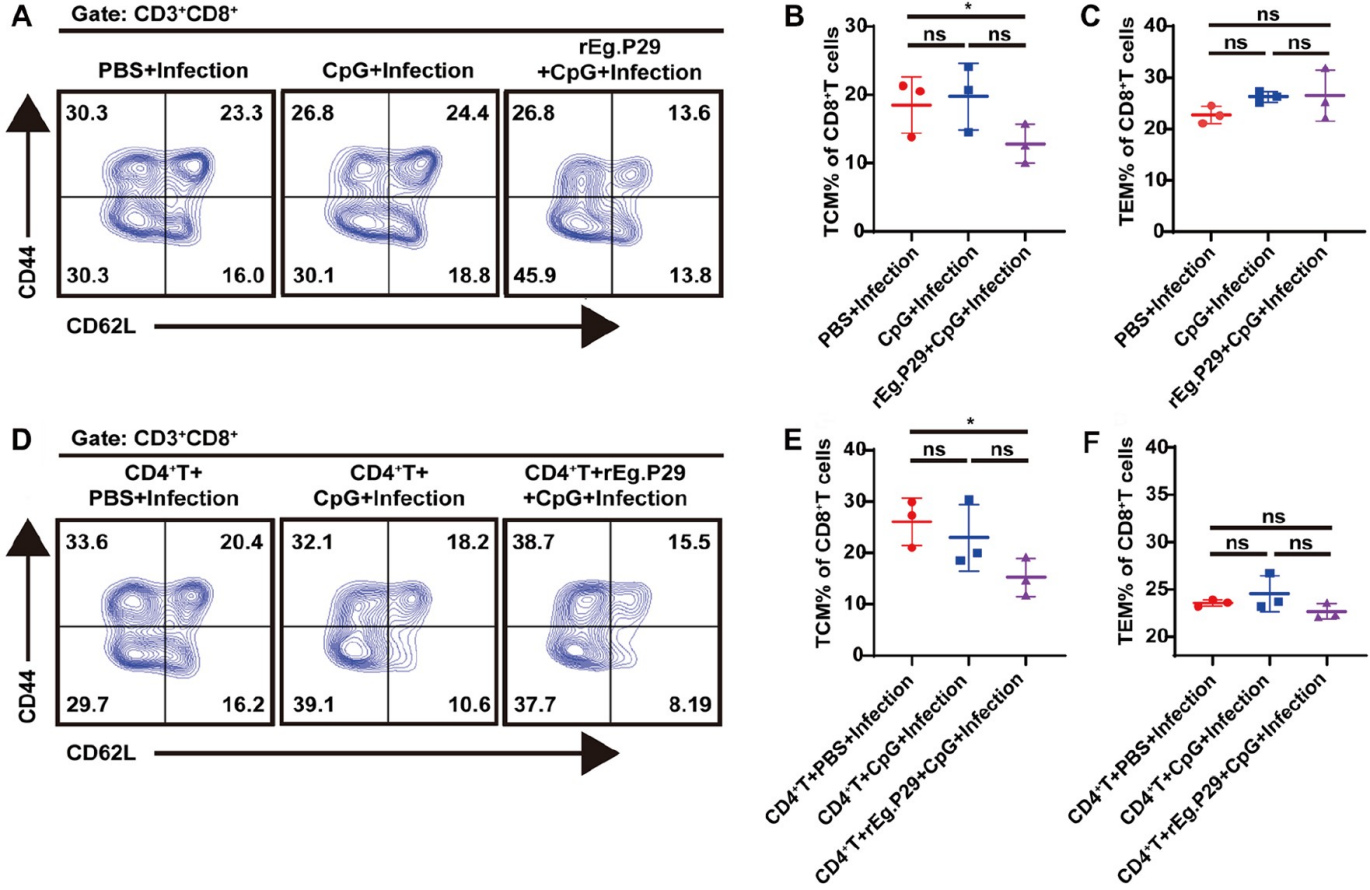
Figure 6 Memory phenotype of CD8 ^+^ T cells At the twelve week after E. multilocularis infection, the spleen was collected, and lymphocytes were isolated. Spleen cells were collected to measure the memory phenotype of CD8+ T cells before or after adoptive transfer of CD4 + T cells from wild-type mice. (A,D) Representative contour plot analysis of gated CD8+ T cells expressing CD44 and CD62L in the spleen. Number of CD8+ T cells presenting the cell surface phenotype (B,E) CD44 +CD62L‒ cells and (C,F) CD44‒CD62L+ cells before or after adoptive transfer of CD4+ T cells. *P<0.05. ns, not significant.

The vaccine induced the formation and maintenance of memory cells, which play a critical role in protecting against parasite infection. Spleen lymphocytes were used to detect CD44 ^‒^CD62L ^+^CD8 ^+^CD8 ^+^ and CD44 ^+^CD62L ^‒^CD8 ^+^memory cells by flow cytometry 12 weeks after priming and boost immunization. As shown in Figure **7**, the proportion of CD44 ^+^CD62L ^‒^CD8 ^+^ memory cells (Tcm) was significantly decreased in the rEg.P29-immunized group compared with that in the PBS or CpG group ( Figure 6A,B), while the proportion of CD44 ^‒^CD62L ^+^CD8 ^+^ memory cells (Tem) was not significantly changed compared that in the PBS and CpG groups ( Figure 6A,C). After adoptive transfer of CD4 ^+^ T cells, the CD44 ^+^CD62L ^‒^CD8 ^+^ T cells of the rEg.P29+Infection group were decreased compared with those of the PBS+Infection group and CpG+Infection group, while the proportions of CD44 ^‒^CD62L ^+^CD8 ^+^ T cells were not significantly different ( Figure 6D‒F).

## Discussion

AE is a zoonotic parasitic disease. Humans are infected by accidental oral uptake of viable eggs, and the eggs parasitize the liver tissue as the blood circulates and form cysts in the liver. The alveolar cyst causes a maling tumor-like lesions with infiltrative, proliferative and destructive character, which locates in the liver primarily, then metastasizes to the other organs. Surgery is the main therapeutic option, but its outcomes are compromised by postoperative complications. Albendazole may have a certain therapeutic effect, but it should be administered continuously and cause toxic side effects; therefore, the current cure rate of AE is only 30%‒40% per year [18].

The incidence of AE is approximately 18,200 cases per year worldwide, resulting in approximately 666,000 disabilities every year, and 91% of new cases occur in China [ 19, 20]. Vaccines are the most economical and effective way to prevent and control infectious diseases [21]. In recent years, studies have suggested that vaccination is an effective way to control and prevent *E* . *multilocularis* infection, and immunization with recombinant proteins of parasites, such as leucine aminopeptidase (LAP), EMY162, EM95 and tetraspanin (TSP), could protect against *E*. *multilocularis* infection and infiltration in host livers [22]. A previous study showed that the REMY162 antigen induced a significant level of protection for the host (74.3%) in experimental infection with *E* . *multilocularis* eggs in mice [23]. Vaccination with rEm-LAP significantly decreased both the number and size of cysts in an *E*. *multilocularis* metacestode-infected mouse model [24]. Our results suggested that the protection of rEg.P29 against CD4 ^+^ T cell-deficient mice with *E*. *multilocularis* was 35.74%, and rEg.P29 could also induce CD4 ^+^ T cell-deficient mice to produce high levels of antibody responses and CD8 ^+^ T-cell immune responses. These results clarified that rEg.P29 induced antibody response and CD8 ^+^ T cell response to prevent *E*. *multilocularis*. The mechanisms of immunity include parasite-neutralizing antibodies that can inhibit parasite motility in the liver at the site of infection and in the bloodstream during transit to the hepatocyte host cell and block the interaction with host cell receptors on hepatocytes [25].

According to the secretion of cytokines, CD4 ^+^ T cells are divided into different subpopulations and play various roles in a large number of diseases, such as Th1, Th2, Th17 and Th9. Th1 cells mainly secrete the cytokine IFN-γ, while the cytokine IL-4 is induced by Th2 lymphocytes. Th17 lymphocytes can secrete IL-17 cytokines [26], while IL-9 is induced by Th9 lymphocytes [27]. During the early stage of *E*. *multilocularis* development, IFN-γ secreted by Th1 lymphocytes is able to eliminate metacestodes at the initial stages of development. With the development of infection, the Th2 immune response gradually becomes dominant in an infective *E*. *multilocularis* mouse model [28]. In the present study, we found that the protection of rEg.P29 against *E* . *multilocularis* was 41.52% after adoptive transfer of CD4 ^+^ T cells from wild-type mice and rEg.P29 induced high levels of IgG, IgM, IgA and IgE antibodies and IFN-γ-secreting CD8 ^+^ T cells, and the number of CD4 ^+^ T cells was significantly increased, but IFN-γ-secreting CD4 ^+^ T cells were not significantly different after adoptive transfer. We infected 2×10 ^6^ CD4 ^+^ T cells from wild-type mice via the tail vein. Only a small portion of CD4 ^+^ T cells resided in the spleen tissue, and the other portion of CD4 ^+^ T cells reached the infection site or other organisms in the blood circulation and played a role in anti- *E*. *multilocularis* infection. In addition, we used bioinformatics to compare the amino acid sequences and protein spatial structure of the P29 proteins of *E*. *multilocularis* and *E*. *granulosus* and found that there are four amino acid mutations between *E*. *granulosus* and *E*. *multilocularis* ( Supplementary Figure S2), and the position of the amino acid mutation may be an antigen epitope and cause the rEg.P29 antigen being unable to recognize the epitope of *E*. *multilocuris*. The accessibility of an epitope combined with the right amino acid sequences to form resistant complexes with MHC class II can establish dominance over the other epitopes by being presented at higher quantities to T cells [29]. Spatial structure comparison showed that the structure of the P29 protein is basically the same between *E*. *granulosus* and *E*. *multilocularis* ( Supplementary Figure S3).

During the vaccine development process, selecting appropriate adjuvants can significantly increase the effectiveness of the vaccine. In our priming and boost schedule, adjuvant CpG ODNs promoted the immunogenicity of rEg.P29. CpG ODNs improve the function of professional antigen-presenting cells and boost the generation of humoral and cellular vaccine-specific immune responses [30]. In our previous research, CpG ODNs were not found to independently induce specific immune responses without rEg.P29, but it can enhance the immune effect without inducing a specific immune response [31].

In summary, the protective effect of rEg.P29 against *E*. *multilocularis* was 35.74% without CD4 ^+^ T cells. After adoptive transfer of CD4 ^+^ T cells from wild mice, the protective effect of rEg.P29 was increased to 41.52%, suggesting that CD4 ^+^ T cells also play critical roles during anti- *E*. *multilocularis* infection.

## Supporting information

466FigS1-S3

## Supplementary Data

Supplementary data is available at *Acta Biochimica et Biphysica Sinica* online.

## References

[1] Gottstein B, Hemphill A (2008). Echinococcus multilocularis: the parasite–host interplay. Exp Parasitology.

[2] Koike A, Becker F, Sennhenn P, et al. Targeting Echinococcus multilocularis PIM kinase for improving anti-parasitic chemotherapy.
PLoS Negl Trop Dis 2022, 16d: e0010483. https://doi.org/10.1371/journal.pntd.0010483.

[3] Khan A, Umhang G, Ullah Z, Boué F, Bastid V, Ullah I, Mahmood S (2021). Investigation of Echinococcus multilocularis in foxes and dogs in Pakistan by detection of copro-DNA. Parasitol Res.

[4] Chong S, Chen G, Dang Z, Niu F, Zhang L, Ma H, Zhao Y (2022). *Echinococcus multilocularis* drives the polarization of macrophages by regulating the RhoA-MAPK signaling pathway and thus affects liver fibrosis. Bioengineered.

[5] Carmena D, Benito A, Eraso E (2007). The immunodiagnosis of Echinococcus multilocularis infection. Clin Microbiol Infect.

[6] Wang Y, Yan Y, Wang Z, Wei X, Wang Z (2023). Rare recurrent brain alveolar echinococcosis complicated by systemic multiorgan infection—a case description. Quant Imag Med Surg.

[7] Pang MQ, Lu YQ, Tang F, Wang HJ, Zhou Y, Ren L, Li RL (2022). Prediction and identification of epitopes in the Echinococcus multilocularis thrombospondin 3 antigen. THC.

[8] Xu X, Qian X, Gao C, Pang Y, Zhou H, Zhu L, Wang Z (2022). Advances in the pharmacological treatment of hepatic alveolar echinococcosis: from laboratory to clinic. Front Microbiol.

[9] Weingartner M, Stücheli S, Jebbawi F, et al. Albendazole reduces hepatic inflammation and endoplasmic reticulum-stress in a mouse model of chronic Echinococcus multilocularis infection.
*
PLoS Negl Trop Dis
* 2022, 16: e0009192. https://doi.org/10.1371/journal.pntd.0009192.

[10] Lightowlers MW, Colebrook AL, Gauci CG, Gauci SM, Kyngdon CT, Monkhouse JL, Vallejo Rodriquez C (2003). Vaccination against cestode parasites: anti-helminth vaccines that work and why. Vet Parasitology.

[11] Gauci C, Merli M, Muller V, Chow C, Yagi K, Mackenstedt U, Lightowlers MW (2002). Molecular cloning of a vaccine antigen against infection with the larval stage of
*Echinococcus multilocularis*. Infect Immun.

[12] Siles-Lucas M, Merli M, Mackenstedt U, Gottstein B (2003). The Echinococcus multilocularis 14-3-3 protein protects mice against primary but not secondary alveolar echinococcosis. Vaccine.

[13] Wang C, Yang SH, Niu N, Tao J, Du XC, Yang JH, Zhu MX (2021). lncRNA028466 regulates Th1/Th2 cytokine expression and associates with Echinococcus granulosus antigen P29 immunity. Parasites Vectors.

[14] Wang H, Li Z, Gao F, Zhao J, Zhu M, He X, Niu N (2016). Immunoprotection of recombinant Eg.P29 against Echinococcus granulosus in sheep. Vet Res Commun.

[15] Gharibi Z, Rahdar M, Pirestani M, Tavalla M, Tabandeh MR (2021). The immunization of protoscolices P29 DNA vaccine on experimental cystic echinococosis in Balb/c mice. Acta Parasit.

[16] Lv Y, Zhu Y, Chang L, Yang J, Zhao Y, Zhao J, Wang Y (2022). Identification of a dominant murine T-cell epitope in recombinant protein P29 from
*Echinococcus granulosus*. Acta Biochim Biophys Sin.

[17] Du X, Zhu M, Zhang T, Wang C, Tao J, Yang S, Zhu Y (2022). The recombinant Eg.p29-mediated miR-126a-5p promotes the differentiation of mouse naive CD4+ T cells via DLK1-mediated notch1 signal pathway. Front Immunol.

[18] Craig P. Echinococcus multilocularis.
*
Curr Opin Infect Dis
* 2003, 16: 437–444. https://doi.org/.

[19] Deplazes P, Rinaldi L, Alvarez Rojas CA, Torgerson PR, Harandi MF, Romig T, Antolova D,
*et al*. Global distribution of alveolar and cystic echinococcosis.
*
Adv Parasitol
* 2017, 95: 315–493. https://doi.org/10.1016/bs.apar.2016.11.001.

[20] Wen H, Vuitton L, Tuxun T, Li J, Vuitton DA, Zhang W, McManus DP (2019). Echinococcosis: advances in the 21st century. Clin Microbiol Rev.

[21] Brisse M, Vrba SM, Kirk N, Liang Y, Ly H (2020). Emerging concepts and technologies in vaccine development. Front Immunol.

[22] Zhou P, Zhou Z, Huayu M, Wang L, Feng L, Xiao Y, Dai Y (2023). A multi-epitope vaccine GILE against Echinococcus multilocularis infection in mice. Front Immunol.

[23] Kouguchi H, Matsumoto J, Katoh Y, Oku Y, Suzuki T, Yagi K (2007). The vaccination potential of EMY162 antigen against Echinococcus multilocularis infection. Biochem Biophys Res Commun.

[24] Wang L, Wei W, Zhou P, Liu H, Yang B, Feng L, Ge RL (2021). Enzymatic characteristics and preventive effect of leucine aminopeptidase against Echinococcus multilocularis. Acta Tropica.

[25] Powell TJ, Tang J, DeRome ME, Mitchell RA, Jacobs A, Deng Y, Palath N (2013). Plasmodium falciparum synthetic LbL microparticle vaccine elicits protective neutralizing antibody and parasite-specific cellular immune responses. Vaccine.

[26] Pang N, Zhang F, Ma X, Zhu Y, Zhao H, Xin Y, Wang S (2014). TGF-β/Smad signaling pathway regulates Th17/Treg balance during echinococcus multilocularis infection. Int Immunopharmacol.

[27] Tuxun T, Apaer S, Ma HZ, Zhang H, Aierken A, Lin RY, Wen H (2015). The potential role of Th9 cell related cytokine and transcription factors in patients with hepatic alveolar echinococcosis. J Immunol Res.

[28] Ma X, Zhang X, Liu J, Liu Y, Zhao C, Cai H, Lei W (2020). The correlations between Th1 and Th2 cytokines in human alveolar echinococcosis. BMC Infect Dis.

[29] Sadegh-Nasseri S, Kim AR (2019). Selection of immunodominant epitopes during antigen processing is hierarchical. Mol Immunol.

[30] Bode C, Zhao G, Steinhagen F, Kinjo T, Klinman DM (2011). CpG DNA as a vaccine adjuvant. Expert Rev Vaccines.

[31] Wørzner K, Sheward DJ, Schmidt ST, Hanke L, Zimmermann J, McInerney G, Karlsson Hedestam GB (2021). Adjuvanted SARS-CoV-2 spike protein elicits neutralizing antibodies and CD4 T cell responses after a single immunization in mice. EBioMedicine.

