# Supplementary material for: Immunoprotective effect and mechanism of rEg.P29 against CD4 + T cell-deficient mice with Echinococcus multilocularis infection : Protection and mechanism of rEg.P29 against E. multilocularis
Source: Acta Biochim Biophys Sin (Shanghai). 2023 Dec 28;56(3):482–9. doi: 10.3724/abbs.2023282 (PMC10984858; doi:10.3724/abbs.2023282)
Supplement: 466FigS1-S3 [file 466FigS1-S3.pdf]

P29 [Echinococcus multilocularis]

Sequence ID: AHA85399.1 Length: **238** Number of Matches: **1**

Range 1: 1 to 238 GenPept Graphics ▼ Next Match ▲ Previous Match

Score	Expect	Method	Identities	Positives	Gaps
480 bits(1236)	7e-180	Compositional matrix adjust.	234/238(98%)	236/238(99%)	0/238(0%)

```
Query  1    MSGFDVTKTFNRFTQRAGELVNKNEKTSYPTRTSDLIHEIDQMKAWISKIITATEEFVDI  60
            MSGFDVTKTFNRFTQRAGELVNKNEKTSYPTRTSDLIHEIDQMKAWISKIITATEEFVDI
Sbjct  1    MSGFDVTKTFNRFTQRAGELVNKNEKTSYPTRTSDLIHEIDQMKAWISKIITATEEFVDI  60

Query  61   NIASKVADAFQKNKEKITTTDKLGTALEQVASQSEKAAPQLSKMLTEASDVHQRMATARK  120
            NIASKV DAFQKNKEKITTTDKLGTALEQVASQSEKAAPQLSKMLTEA+DVHQRMATARK
Sbjct  61   NIASKVVDAFQKNKEKITTTDKLGTALEQVASQSEKAAPQLSKMLTEAADVHQRMATARK  120

Query  121  NFNSEVNTTFIEDLKNFLNTTLSEAQKAKTKLEEVRLDLDSDKTKLKNAKTAEQKAKWEA  180
            NFNSEVNTTFIEDLKNFLNTTLSEAQKAKTKLEEVRLDLDSDKTKLKNAKTAEQKAKWEA
Sbjct  121  NFNSEVNTTFIEDLKNFLNTTLSEAQKAKTKLEEVRLDLDSDKTKLKNAKTAEQKAKWEA  180

Query  181  EVRKDESDFDRVHQESLTIFEKTCKEFDGLSVQLLDLIRAEKNYYEACAKECSMMLGE  238
            EVRKDESDFDRVHQESL +FEKTCKEFDGLSVQLLDLIRAEKNYYEACAKECSMMLGE
Sbjct  181  EVRKDESDFDRVHQESLAVFEKTCKEFDGLSVQLLDLIRAEKNYYEACAKECSMMLGE  238
```

Supplementary Figure S1. P29 sequences were blasted between *Echinococcus granulosus* and *Echinococcus multilocularis* The sequences of Eg.P29 and Em.P29 were obtained from NCBI database and run blast.

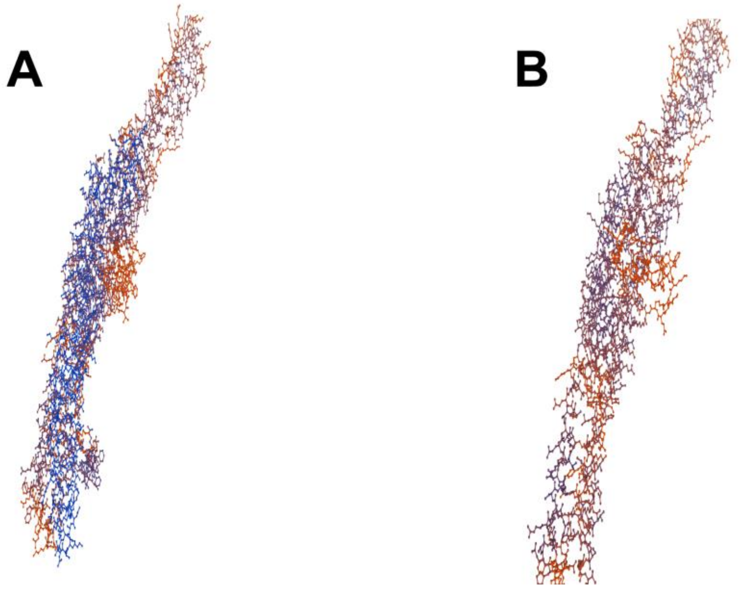

Supplementary Figure S2. The spatial structures of Eg.P29 and Em.P29

SWISS-MODEL was used to predict the spatial structure of Eg.P29 and Em.P29. (A) The structure of Eg.P29. (B) The structure of Em.P29.

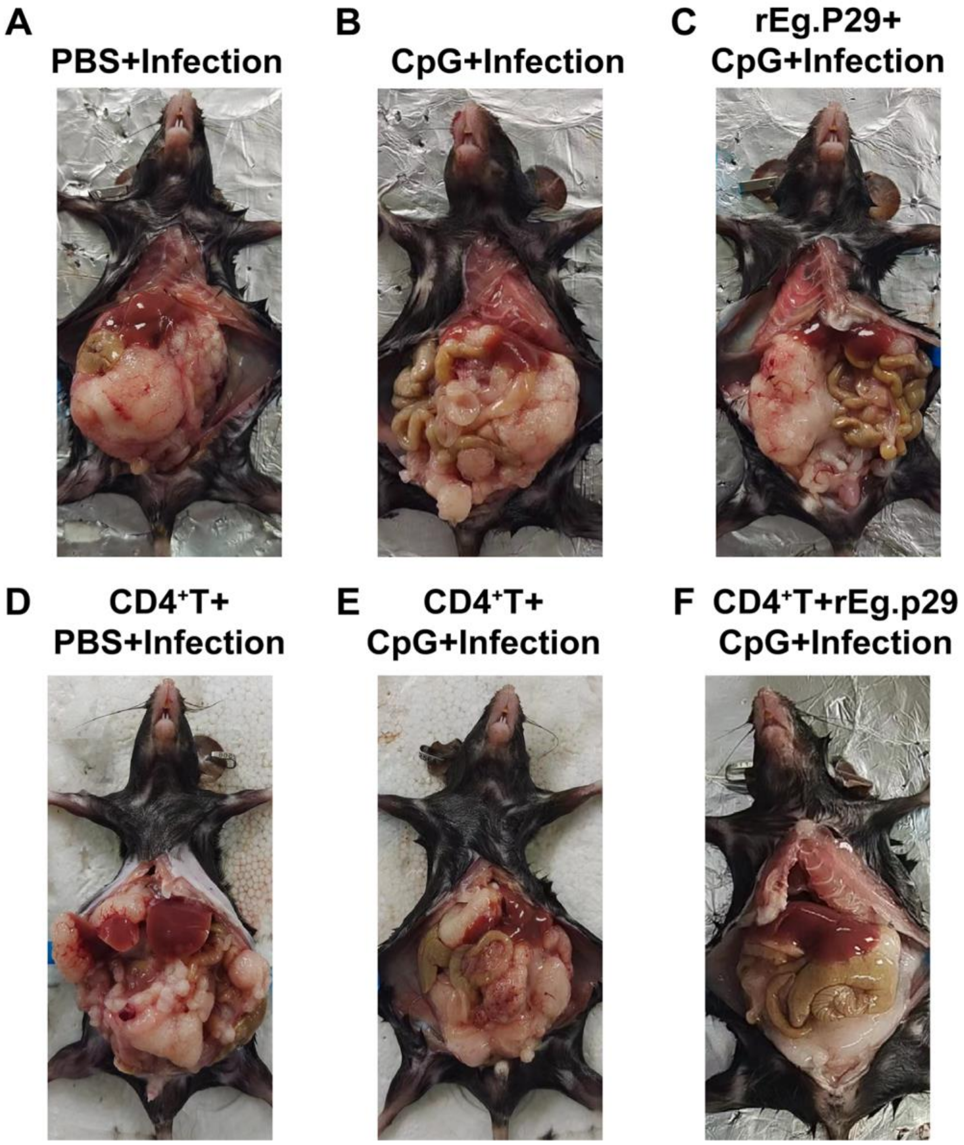

Supplementary Figure S3. Anatomical view of intraperitoneally infected protoscolex mice in each group At the 12th week after *E. multilocularis* infection, the mice were anaesthetized with isoflurane, and the infection status of *Echinococcus multilocularis* in the abdominal cavity of mice in each group was observed.
